# The risk of false inclusion of a relative in parentage testing – an *in silico* population study

**DOI:** 10.3325/cmj.2013.54.257

**Published:** 2013-06

**Authors:** James Chun-I Lee, Li-Chin Tsai, Pao-Ching Chu, Yen-Yang Lin, Chun-Yen Lin, Tsun-Ying Huang, Yu-Jen Yu, Adrian Linacre, Hsing-Mei Hsieh

**Affiliations:** 1 Department of Forensic Medicine, College of Medicine, National Taiwan University, Taipei, Taiwan ROC; 2 Institute of Forensic Medicine, Ministry of Justice, New Taipei City, Taiwan ROC; 3 Department of Forensic Science, Central Police University, Taoyuan, Taiwan ROC; 4 School of Biological Sciences, Flinders University, Adelaide, Australia

## Abstract

**Aim:**

To investigate the potential of false inclusion of a close genetic relative in paternity testing by using computer generated families.

**Methods:**

10 000 computer-simulated families over three generations were generated based on genotypes using 15 short tandem repeat loci. These data were used in assessing the probability of inclusion or exclusion of paternity when the father is actually a sibling, grandparent, uncle, half sibling, cousin, or a random male. Further, we considered a duo case where the mother’s DNA type was not available and a trio case including the mother’s profile.

**Results:**

The data showed that the duo scenario had the highest and lowest false inclusion rates when considering a sibling (19.03 ± 0.77%) and a cousin (0.51 ± 0.14%) as the father, respectively; and the rate when considering a random male was much lower (0.04 ± 0.04%). The situation altered slightly with a trio case where the highest rate (0.56 ± 0.15%) occurred when a paternal uncle was considered as the father, and the lowest rate (0.03 ± 0.03%) occurred when a cousin was considered as the father. We also report on the distribution of the numbers for non-conformity (non-matching loci) where the father is a close genetic relative.

**Conclusions:**

The results highlight the risk of false inclusion in parentage testing. These data provide a valuable reference when incorporating either a mutation in the father’s DNA type or if a close relative is included as being the father; particularly when there are varying numbers of non-matching loci.

The use of an increasing number of loci in a multiplex amplification leads inevitably to higher confidence in assignment of an individual as being a defined genetic relative of a known person. With an increase in the loci used in a paternity test comes also the increase in the chance of observing a mutational event; leading to the possibility of a false exclusion. However, there also comes the benefit of a potential higher power of discrimination. When testing close genetic relatives as part of a paternity assignment, it is expected that more alleles will be shared, such as in full siblings (1), when compared to a random member of the population. In support of this assumption, a previous study indicated that there was at least a 50% chance of two random men sharing at least one allele at 10 of the 14 loci tested (2). The chance of a false inclusion and exclusion is greater when testing one putative parent and an offspring (a duo scenario) than when there is an additional confirmed parent (a trio scenario). In instances of immigration cases, it may be that one relative poses as a parent of a child; such an incident was reported when a sibling claimed to be the father of a boy (3). The instance when a close genetic relative posed as a parent of an offspring where 9 or 10 loci were used in a paternity test led to unsatisfactory results (4). A similar study highlighted an instance when using 11 polymorphic short tandem repeat (STR) loci there was a matching allele at each locus between a child, the assumed mother, and skeletal remains that were not from the father of that child (5); this same study found 3 further instances of exactly the same scenario when using 10 STR loci. Recently, there has been a report of two tested men presenting matching alleles with a potential offspring at 19 STR loci in a duo case (6).

The probability of excluding a relative from being a true father of an offspring was examined using data for 12 STR loci from a known population (7). An extension of this study, using 12 STR loci, derived the probability of excluding a relative for close genetic relatives (8). A conclusion was that full siblings impersonating parent/child proved the most difficult scenario to discredit with DNA profiling alone. Similarly, it was reported that there was a probability of 12% that there would be no inconsistencies (a shared allele at all loci tested) when comparing data using 18 STR loci when a sibling of a true parent posed as the parent of the tested child (9). In motherless paternity analysis using 15 STR loci, the differences between probabilities for father and uncle were observed to be small (10).

The use of computer-simulated populations has the great benefit of an increase in the size of the available data. Evaluation of the efficacy of trio sibship testing and sibling assignment for forensic purposes by using such model populations was performed in our laboratory (11). In this study, we report on the false paternity probabilities with 15 STR loci when comparing two close genetic relatives (two siblings, paternal grandparent/grandchild, paternal uncle/nephew or niece, two half siblings, and two cousins) and two random persons. These different combinations were generated using 10 000 simulated 3-generation families based on data from the Taiwan population (12). The risks of false inclusion for duos and trios in parentage testing were evaluated respectively.

## Materials and methods

### Populations

**Figure 1:**
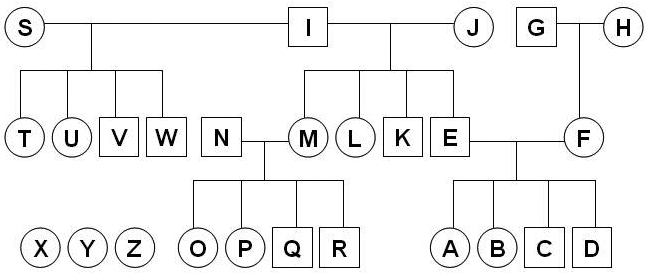
The pedigree of the family used where circles indicate women and squares men. Three random persons are included (X, Y, and Z).

A total of 10 000 family groups extending over 3 generations were simulated using 15 STR loci. These data were created using allele frequencies from the study of Lee et al (12). In this previous study, allele frequencies were calculated from 3794 random individuals of Taiwanese Han Population using the software PowerMarker (*http://statgen.ncsu.edu/powermarker/index.html*). The 15 STR loci were analyzed by using the AmpFlSTR® Identifiler PCR Amplification Kit (Applied Biosystems, Foster City, CA, USA). Genotypes of members G, H, I, J, N, S, X, Y, and Z were randomly generated and those of their off-springs were generated following Mendel’s laws of inheritance in a spreadsheet of Microsoft® Office Excel 2007 using functions “countif,” “indirect,” “address,” “if,” and “randbetween.” The potential for a mutational event was not taken in account while creating family groups. The duo/trio populations, each with 10 000 combinations, were established with combinations of EB/EFB (duo/trio or true parents), CB/CFB (sibling as the father), IB/IFB (paternal grandfather as the father), KB/KFB (paternal uncle as the father), WM/WJM (half-sibling, child’s half brother, as the father), RB/RFB (cousin, being the son of father’s sister, as the father), and XB/XFB (random male as the father) (Figure 1).

### Calculations

The STR genotypes were entered into a spreadsheet and all calculations were performed using Microsoft Office Excel 2007. The likelihood ratio (LR) (or paternity index) of duo and trio parentage testing was calculated using the algorithm recommended by the ISFG (13), where the numerator assumes the tested man is the father and the denominator assumes a random man is the father. A value of zero was used for the non-matching loci. The confidence intervals for a proportion of non-exclusion rates were calculated with Modified Wald method (Agresti-Coull Interval) (14).

## Results

### Possible false inclusions in duo cases

This study was designed to illustrate the potential of a misinterpretation of paternal relative (such as a grandparent, uncle, sibling, half sibling, and cousin) being a biological father compared to a random man in paternity testing. In duo cases, the highest non-exclusion rate was 19.03 ± 0.77% in the scenario where a sibling posed as the father (Table 1, eg, CB in Figure 1). This indicated that in 19.03 ± 0.77% cases, the child’s sibling could not be distinguished from the true father. The non-exclusion rates when other relatives posed as the father were 2.81 ± 0.32% (grandparent-child, eg, IB), 2.78 ± 0.32% (uncle-child, eg, KB), 2.58 ± 0.31% (half sibling-child, eg, WM), and 0.51 ± 0.14% (cousin-child, eg, RB). The combination with the highest non-exclusion rate was when a sibling posed as the father. In this scenario, the accumulative non-exclusion rate was as high as 51.7 ± 0.98% when assuming one non-matching locus was due to a mutation.

**Table 1:** The distribution of the numbers for non-conformity based on 15 loci and accumulative non-exclusion rates for each of the close relatives and random man as alleged father in duo parentage testing*

Relative		Non-conformity number															
		0	1	2	3	4	5	6	7	8	9	10	11	12	13	14	15
Parent	I	10000	0	0	0	0	0	0	0	0	0	0	0	0	0	0	0
	II	10000	10000	10000	10000	10000	10000	10000	10000	10000	10000	10000	10000	10000	10000	10000	10000
	III	99.98 ± 0.03	99.98 ± 0.03	99.98± 0.03	99.98± 0.03	99.98 ± 0.03	99.98± 0.03	99.98± 0.03	99.98± 0.03	99.98± 0.03	99.98± 0.03	99.98± 0.03	99.98± 0.03	99.98± 0.03	99.98± 0.03	99.98± 0.03	99.98± 0.03
Sibling	I	1902	3268	2790	1400	493	121	20	6	0	0	0	0	0	0	0	0
	II	1902	5170	7960	9360	9853	9974	9994	10000	10000	10000	10000	10000	10000	10000	10000	10000
	III	19.03 ± 0.77	51.7± 0.98	79.59 ±0.79	93.58± 0.48	98.51± 0.24	99.72± 0.10	99.92± 0.06	99.98± 0.03	99.98± 0.03	99.98± 0.03	99.98± 0.03	99.98± 0.03	99.98± 0.03	99.98± 0.03	99.98± 0.03	99.98± 0.03
Grand-parent	I	279	1137	2063	2582	1987	1185	546	164	43	10	3	1	0	0	0	0
	II	279	1416	3479	6061	8048	9233	9779	9943	9986	9996	9999	10000	10000	10000	10000	10000
	III	2.81± 0.32	14.17 ± 0.68	34.80± 0.93	60.61± 0.96	80.47± 0.78	92.31± 0.52	97.77± 0.29	99.41± 0.15	99.84± 0.08	99.94± 0.05	99.97± 0.03	99.98± 0.03	99.98± 0.03	99.98± 0.03	99.98± 0.03	99.98± 0.03
Uncle	I	276	1162	2127	2545	2015	1142	514	170	43	6	0	0	0	0	0	0
	II	276	1438	3565	6110	8125	9267	9781	9951	9994	10000	10000	10000	10000	10000	10000	10000
	III	2.78± 0.32	14.39 ± 0.69	35.66± 0.94	61.10± 0.96	81.24± 0.77	92.65± 0.51	97.79± 0.29	99.49± 0.14	99.92± 0.06	99.98± 0.03	99.98± 0.03	99.98± 0.03	99.98± 0.03	99.98± 0.03	99.98± 0.03	99.98± 0.03
Half-sibling	I	256	1148	2110	2567	1980	1205	498	180	46	8	2	0	0	0	0	0
	II	256	1404	3514	6081	8061	9266	9764	9944	9990	9998	10000	10000	10000	10000	10000	10000
	III	2.58± 0.31	14.05 ± 0.68	35.15± 0.94	60.81± 0.96	80.60± 0.78	92.64± 0.51	97.62± 0.30	99.42± 0.15	99.88± 0.07	99.96± 0.04	99.98± 0.03	99.98± 0.03	99.98± 0.03	99.98± 0.03	99.98± 0.03	99.98± 0.03
Cousin	I	49	209	726	1456	2067	2206	1762	909	410	154	43	8	1	0	0	0
	II	49	258	984	2440	4507	6713	8475	9384	9794	9948	9991	9999	10000	10000	10000	10000
	III	0.51± 0.14	2.60± 0.31	9.86± 0.58	24.41± 0.84	45.07± 0.98	67.12± 0.92	84.74± 0.70	93.82± 0.47	97.92± 0.28	99.46 ± 0.14	99.89± 0.06	99.97± 0.03	99.98± 0.03	99.98± 0.03	99.98± 0.03	99.98± 0.03
Random man	I	2	32	133	432	991	1694	2113	1948	1447	762	321	97	23	3	2	0
	II	2	34	167	599	1590	3284	5397	7345	8792	9554	9875	9972	9995	9998	10000	10000
	III	0.04± 0.04	0.36± 0.12	1.69± 0.25	6.01± 0.47	15.91± 0.72	32.85± 0.92	53.97± 0.98	73.44± 0.87	87.90± 0.64	95.52± 0.41	98.73± 0.22	99.70± 0.11	99.93± 0.05	99.96± 0.04	99.98± 0.03	99.98± 0.03

*Each relationship is analyzed by using 10 000 combinations. The 1st line (I) and 2nd line (II) represent the counts and accumulative counts respectively. The non-exclusion rate (III, %) is calculated with Modified Wald Method (Agresti-Coull Interval) and 95% confidence interval.

The Log LR (Logarithmic value of likelihood ratio) for the true parent-child pairs ranged from 1.4845 to 11.4087 (Table 2). For paternity testing, LR reflects how many times more likely the alleged father is to be the child’s father than any male taken at random from the population. The mean value of Log LR (α = 0.05) was similar when comparing the true parent-child pairs (5.0207 ± 0.0247) to the sibling-child pairs (5.6010 ± 0.0577). It should be noted that the mean value of Log LR for the sibling-child pairs was even higher than the true parent-child pairs; however the standard deviation for the sibling-child pairs (0.0577) was higher than the true parent-child pairs (0.0247).

**Table 2:** The distribution of logarithmic value of likelihood ratio (Log LR) for each of the close relatives and random man as alleged father in duo parentage testing based on 10 000 computer generated families’ genotypes

		Log LR (Probability of paternity, %)*		
Relative	No. of non- exclusion pairs	Minimum	Maximum	Mean ± standard deviation (α = 0.05)
Parent	10000	1.4845 (96.8268)	11.4087 (100.0000)	5.0207 ± 0.0247 (99.9990)
Sibling	1902	1.2337 (94.4836)	10.4002 (100.0000)	5.6010 ± 0.0577 (99.9997)
Grandparent	279	1.5582 (97.3088)	7.9826 (100.0000)	4.0263 ± 0.1346 (99.9906)
Uncle	276	1.6478 (97.7994)	8.5425 (100.0000)	4.2644 ± 0.1412 (99.9946)
Half-sibling	256	1.0975 (92.6019)	6.8732 (100.0000)	4.2150 ± 0.1343 (99.9939)
Cousin	49	0.7880 (85.9897)	6.1611 (99.9999%)	3.4479 ± 0.3127 (99.9644)
Random man	2	2.8206 (99.8491)	3.2468 (99.9434)	3.0337 ± 2.7075 (99.9076)

*Probability of paternity – calculated by LR/(LR + 1).

### Possible false inclusions in trio cases

In trio cases, the highest non-exclusion rate was 0.56 ± 0.15% in the scenario where a paternal uncle posed as the father (Table 3, eg, KFB in Figure 1). The non-exclusion rates in scenarios where other relatives posed as the child’s father were 0.51 ± 0.14% (sibling, eg, CFB), 0.46 ± 0.13% (half sibling, eg, WJM), 0.38 ± 0.12% (grandparent, eg, IFB), and 0.03 ± 0.03% (cousin, eg, RFB) for each of the 10 000 combinations. The highest non-exclusion rate was observed in the case of the uncle posing as the father, where the accumulative non-exclusion rate for this relationship was 3.57 ± 0.36% assuming a mutation; however, under this scenario, it was highest for the sibling relationship (4.28 ± 0.40%).

**Table 3:** The distribution of the numbers for non-conformity in 15 loci and accumulative non-exclusion rates for each of the close relatives and random man as alleged father in trio parentage testing*

Relative		Non-conformity number															
		0	1	2	3	4	5	6	7	8	9	10	11	12	13	14	15
Parent	I	10000	0	0	0	0	0	0	0	0	0	0	0	0	0	0	0
	II	10000	10000	10000	10000	10000	10000	10000	10000	10000	10000	10000	10000	10000	10000	10000	10000
	III	99.98 ± 0.03	99.98 ± 0.03	99.98± 0.03	99.98± 0.03	99.98± 0.03	99.98± 0.03	99.98± 0.03	99.98± 0.03	99.98± 0.03	99.98± 0.03	99.98± 0.03	99.98± 0.03	99.98± 0.03	99.98± 0.03	99.98± 0.03	99.98± 0.03
Uncle	I	54	301	935	1763	2210	2039	1502	752	303	106	26	9	0	0	0	0
	II	54	355	1290	3053	5263	7302	8804	9556	9859	9965	9991	10000	10000	10000	10000	10000
	III	0.56± 0.15	3.57± 0.36	12.91± 0.66	30.54± 0.90	52.63± 0.98	73.01± 0.87	88.02± 0.64	95.54± 0.40	98.57± 0.23	99.63± 0.12	99.89± 0.06	99.98± 0.03	99.98± 0.03	99.98± 0.03	99.98± 0.03	99.98± 0.03
Sibling	I	49	377	1063	1879	2244	2016	1326	655	271	91	25	4	0	0	0	0
	II	49	426	1489	3368	5612	7628	8954	9609	9880	9971	9996	10000	10000	10000	10000	10000
	III	0.51± 0.14	4.28± 0.40	14.90± 0.70	33.69± 0.93	56.12± 0.97	76.27± 0.83	89.52± 0.60	96.07± 0.38	98.78± 0.22	99.69± 0.11	99.94± 0.05	99.98± 0.03	99.98± 0.03	99.98± 0.03	99.98± 0.03	99.98± 0.03
Half-sibling	I	44	306	915	1693	2302	2071	1518	734	291	101	23	2	0	0	0	0
	II	44	350	1265	2958	5260	7331	8849	9583	9874	9975	9998	10000	10000	10000	10000	10000
	III	0.46± 0.13	3.52± 0.36	12.66± 0.65	29.59± 0.89	52.60± 0.98	73.30± 0.87	88.47± 0.63	95.81± 0.39	98.72± 0.22	99.73± 0.10	99.96± 0.04	99.98± 0.03	99.98± 0.03	99.98± 0.03	99.98± 0.03	99.98± 0.03
Grand-parent	I	36	315	939	1668	2170	2114	1489	808	337	89	30	5	0	0	0	0
	II	36	351	1290	2958	5128	7242	8731	9539	9876	9965	9995	10000	10000	10000	10000	10000
	III	0.38± 0.12	3.53± 0.36	12.91± 0.66	29.59± 0.89	51.28± 0.98	72.41± 0.88	87.30± 0.65	95.37± 0.41	98.74± 0.22	99.63± 0.12	99.93± 0.05	99.98± 0.03	99.98± 0.03	99.98± 0.03	99.98± 0.03	99.98± 0.03
Cousin	I	1	20	97	318	836	1457	1944	1985	1649	1020	471	147	45	10	0	0
	II	1	21	118	436	1272	2729	4673	6658	8307	9327	9798	9945	9990	10000	10000	10000
	III	0.03± 0.03	0.23± 0.09	1.20± 0.21	4.38± 0.40	12.73± 0.65	27.30± 0.87	46.73± 0.98	66.57± 0.92	83.06± 0.74	93.25± 0.49	97.96± 0.28	99.43± 0.15	99.88± 0.07	99.98± 0.03	99.98± 0.03	99.98± 0.03
Rand- om man	I	0	1	3	14	82	245	655	1251	1913	2091	1818	1160	548	177	39	3
	II	0	1	4	18	100	345	1000	2251	4164	6255	8073	9233	9781	9958	9997	10000
	III	0.02± 0.03	0.03± 0.03	0.06± 0.05	0.20± 0.09	1.02± 0.20	3.47± 0.36	10.02± 0.59	22.52± 0.82	41.64± 0.97	62.54± 0.95	80.72± 0.77	92.31± 0.52	97.79± 0.29	99.56± 0.13	99.95± 0.04	99.98± 0.03

*Each relationship is analyzed by using 10 000 combinations. The 1st line (I) and 2nd line (II) represent the counts and accumulative counts respectively. The non-exclusion rate (III, %) is calculated with Modified Wald Method (Agresti-Coull Interval) and 95% confidence interval.

The Log LR for the actual parent-mother-child pairs ranged from 4.0061 to 16.0957 (Table 4). The mean value (7.1267) of Log LR (α = 0.05) for the half-sibling-mother-child pairs was the closest value compared to the actual parent-mother-child pairs (7.4741); however, its standard deviation was highest when compared to other combinations of relatives.

**Table 4:** The distribution of logarithmic value of likelihood ratio (Log LR) for each of the close relatives and random man as alleged father in trio parentage testing based on 10 000 computer generated families’ genotypes

		Log LR (Probability of paternity, %)*		
Relative	No. of non- exclusion pairs	Minimum	Maximum	Mean ± standard deviation (α = 0.05)
Parent	10000	4.0061 (99.9901)	16.0957 (100.0000)	7.4741 ± 0.0266 (100.0000)
Uncle	54	4.1748 (99.9933)	9.3911 (100.0000)	6.7822 ± 0.2991 (100.0000)
Sibling	49	4.9335 (99.9988)	9.9194 (100.0000)	6.9927 ± 0.3519 (100.0000)
Half-sibling	44	4.9754 (99.9989)	10.4723 (100.0000)	7.1267 ± 0.4025 (100.0000)
Grandparent	36	5.0455 (99.9991)	8.6544 (100.0000)	6.7147 ± 0.3326 (100.0000)
Cousin	1	7.6185 (100.0000)	7.6185 (100.0000)	-
Random man	0	-	-	-

*Probability of paternity is calculated by LR/(LR + 1).

## Discussion

For the duo cases, the results illustrated the highest non-exclusion rate when a sibling posed as the child’s father. This scenario was in line with a previous report where the most difficult combination to distinguish was when a brother claimed to be the actual father of his sibling and when the mother’s genotype was unavailable (8). It was also noted in this paper that the probability of not excluding a brother as being the father of his sibling using 12 STR loci was about 27%; and if one mismatch was assumed, it increased to 65%, further illustrating the difficulty of excluding a brother as being the father of a sibling.

It has also been reported that if the alleged parent and child are actually uncle and nephew, the probability of excluding a relative was 0.903 based on 9 STR loci in motherless cases (4), rising to 0.937 when 12 common STR loci were used; to 0.966 and 0.984 using 9 and 12 STRs, respectively, when the mother’s genotypes were used. This same study also showed that when 20 STR loci were used, the corresponding probability of excluding a relative was 0.9986 (for a trio) and 0.9875 (for a duo), supporting the assumption that the number of STR markers typed and the inclusion of data from the mother’s profile affected the rates of false inclusion. In this study, 15 STR loci were used.

It was reported by Poetsch et al that no STR mismatches for 15 STR loci between a child and an unrelated man were detected in 26 comparisons (duo cases) out of 116 004 from a region of northern Germany (15). Such a study highlights the opportunity for a false inclusion of paternity when a close genetic relative claims to be the father of a child, especially in a small geographical region.

Even with these data, the access to the genotypes of close relatives remains the preferred option to minimize the chance of a false inclusion; although it should be noted that these data are not always available. In the current study, we report on the risk of false inclusion in parentage testing to provide a valuable reference for forensic laboratories when incorporating either a mutation in the DNA profile from a putative father or when a close relative is the potential father.

We report on the evaluation of possible false inclusions in duo and trio cases when replacing the real/true father with the other close relatives and also with a random man. The highest non-exclusion rates for the duo cases were observed in the scenario where a sibling claimed to be the true father. For the trio cases the highest non-exclusion occurred when a paternal uncle posed as the biological father. When a single mutational event was incorporated into the 15 STR loci test, the highest accumulative non-exclusion rate was observed when a sibling posed as a true father in the duo and trio combinations. The results highlight the risk of potential false inclusion in parentage testing.
